# Therapeutic Effect of Platelet-Rich Plasma in Rat Spinal Cord Injuries

**DOI:** 10.3389/fnins.2018.00252

**Published:** 2018-04-23

**Authors:** Nan-Fu Chen, Chun-Sung Sung, Zhi-Hong Wen, Chun-Hong Chen, Chien-Wei Feng, Han-Chun Hung, San-Nan Yang, Kuan-Hao Tsui, Wu-Fu Chen

**Affiliations:** ^1^Division of Neurosurgery, Department of Surgery, Kaohsiung Armed Forces General Hospital, Kaohsiung, Taiwan; ^2^Department of Neurological Surgery, Tri-Service General Hospital, National Defense Medical Center, Taipei, Taiwan; ^3^Department of Anesthesiology, Taipei Veterans General Hospital, Taipei, Taiwan; ^4^School of Medicine, National Yang-Ming University, Taipei, Taiwan; ^5^Doctoral Degree Program in Marine Biotechnology, National Sun Yat-Sen University, Kaohsiung, Taiwan; ^6^Department of Marine Biotechnology and Resources, National Sun Yat-Sen University, Kaohsiung, Taiwan; ^7^Doctoral Degree Program in Marine Biotechnology, Academia Sinica, Taipei, Taiwan; ^8^School of Medicine, College of Medicine and Department of Pediatrics, E-DA Hospital, I-Shou University, Kaohsiung, Taiwan; ^9^Department of Obstetrics and Gynecology, Kaohsiung Veterans General Hospital, Kaohsiung, Taiwan; ^10^Department of Obstetrics and Gynecology and Institute of Clinical Medicine, National Yang-Ming University, Taipei, Taiwan; ^11^Department of Biological Science, National Sun Yat-Sen University, Kaohsiung, Taiwan; ^12^Department of Pharmacy and Master Program, College of Pharmacy and Health Care, Tajen University, Pingtung County, Taiwan; ^13^Department of Neurosurgery, Kaohsiung Chang Gung Memorial Hospital and Chang Gung University College of Medicine, Kaohsiung, Taiwan; ^14^Department of Neurosurgery, Xiamen Chang Gung Hospital, Fujian, China

**Keywords:** platelet-rich plasma, spinal cord injury, inflammation, angiogenesis, neuroregeneration, rats

## Abstract

Platelet-rich plasma (PRP) is prepared by centrifuging fresh blood in an anticoagulant state, and harvesting the platelet-rich portion or condensing platelets. Studies have consistently demonstrated that PRP concentrates are an abundant source of growth factors, such as platelet-derived growth factor (PDGF), transforming growth factor β (TGF-β), insulin-like growth factor 1 (IGF-1), and epithelial growth factor (EGF). The complex mechanisms underlying spinal cord injury (SCI) diminish intrinsic repair and neuronal regeneration. Several studies have suggested that growth factor-promoted axonal regeneration can occur for an extended period after injury. More importantly, the delivery of exogenous growth factors contained in PRP, such as EGF, IGF-1, and TGF-β, has neurotrophic effects on central nervous system (CNS) injuries and neurodegenerative diseases. However, only a few studies have investigated the effects of PRP on CNS injuries or neurodegenerative diseases. According to our review of relevant literature, no study has investigated the effect of intrathecal (i.t.) PRP injection into the injured spinal cord and activation of intrinsic mechanisms. In the present study, we directly injected i.t. PRP into rat spinal cords and examined the effects of PRP on normal and injured spinal cords. In rats with normal spinal cords, PRP induced microglia and astrocyte activation and PDGF-B and ICAM-1 expression. In rats with SCIs, i.t. PRP enhanced the locomotor recovery and spared white matter, promoted angiogenesis and neuronal regeneration, and modulated blood vessel size. Furthermore, a sustained treatment (a bolus of PRP followed by a 1/3 dose of initial PRP concentration) exerted more favorable therapeutic effects than a single dose of PRP. Our findings suggest by i.t. PRP stimulate angiogenesis, enhancing neuronal regeneration after SCI in rats. Although PRP induces minor inflammation in normal and injured spinal cords, it has many advantages. It is an autologous, biocompatible, nontoxic material that does not result in a major immune response. In addition, based on its safety and ease of preparation, we hypothesize that PRP is a promising therapeutic agent for SCI.

## Introduction

Platelet-rich plasma (PRP) is a concentrated source of autologous platelets in plasma. PRP had beneficial effects in sports injury (Jassim and Haddad, 2012), wound healing (Naik et al., 2013), and after plastic surgery (Sommeling et al., 2013) because it contains autologous growth factors that accelerate tissue healing. Studies have consistently shown that PRP concentrates are an abundant source of growth factors, such as platelet-derived growth factor (PDGF), transforming growth factor β (TGF-β), vascular endothelial growth factor A (VEGF-A), insulin-like growth factor 1 (IGF-1), granulocyte-macrophage colony-stimulating factor (GM-CSF), hepatocyte growth factor (HGF), and epithelial growth factor (EGF) (Pallua et al., 2010; Middleton et al., 2012; Andia and Maffulli, 2013). PRP is prepared by centrifuging fresh blood in an anticoagulant state, and harvesting the platelet-rich portion or condensing platelets. The addition of autologous thrombin and CaCl_2_ triggers the activation process, and once PRP is activated, growth factors begin to be secreted within 10 min; about 95% of all growth factors are secreted in 1 h (Arora et al., 2009). Suggesting by control the concertation of PRP would control the amount of growth factors.

Current spinal cord injury (SCI) research is focused on limiting secondary injury mechanisms including apoptosis, inflammation, excitotoxicity, and reactive oxygen species (ROS) generation. The promotion of neuronal regeneration is less addressed, although recent evidence suggests that providing a permissive surrounding for axonal growth is beneficial for axon regeneration after SCI (Lu et al., 2012). Functional recovery is limited because axons in the central nervous system (CNS) fail to regenerate. After SCI, there is a lack of suitable substrates to support axonal attachment and extension through the lesion site (Bunge, 2001), a lack of growth factors to activate neuron-intrinsic regenerative programs (Jones et al., 2001), and secondary damage resulting from inflammatory mechanisms (Jones et al., 2005).

More importantly, the delivery of exogenous growth factors contained in PRP, such as EGF, IGF, and TGF-β, has been shown to have neurotrophic effects in CNS injuries and neurodegenerative diseases (Flanders et al., 1998; Wang et al., 2000; Kojima and Tator, 2002). The growth factors and neurotropic factors such as VEGF, IGF, PDGF, and EGF in PRP had therapeutic effects in CNS injuries. Therefore, many articles believed that PRP had therapeutic effects in CNS injury. However, only few reports have studied the effects of PRP on CNS injuries or neurodegenerative diseases. Recent research on the use of PRP to treat neurologic diseases has focused on peripheral nerve injuries, such as sciatic nerve injury (Lichtenfels et al., 2013), cavernous nerve injury (Wu et al., 2016), and facial nerve injury (Cho et al., 2010), and peripheral neuropathy (Anjayani et al., 2014). The reports of PRP relative to SCI were used PRP gel (activated PRP) as a scaffold to seed brain-derived neurotropic factor-overexpressing bone marrow stromal cell in the hemi-section SCI animal model (Zhao et al., 2013), and the another research used brain-spinal cord culture (Takeuchi et al., 2012) to examine the neuronal generation after PRP treatment. However, to the best of our knowledge there is no research of intrathecal (i.t.) injection PRP into injured spinal cord and examine the effect of activation of intrinsic mechanisms. In the present study, we studied the effects of i.t. PRP in normal or SCI rats to examine the possible therapeutic effects of PRP in SCI.

## Materials and methods

### Implantation of i.t. catheters and spinal cord contusion injury

The implantation of i.t. catheters and induction of spinal cord contusion injuries were performed as previously described (Chen et al., 2015, 2016). Female Wistar rats (10 weeks old) were used in this study. In brief, i.t. catheters were implanted into the i.t. space at the T12 level of the spinal cord through the atlanto-occipital membrane. A 4-day recovery period was allowed after the i.t. catheter implantation. Under 2.5% isoflurane anesthesia, the thoracic area of the rats was shaved following the application of iodine to the skin. Laminectomy of the caudal portion of T9 and all of T10 was performed to expose the spinal cord. The intact T10 spinal cord area was contused with the New York University (NYU) impactor (W.M. Keck Center for Collaborative Neuroscience, Rutgers University, New Brunswick, NJ, USA) weight-drop device (from a height of 50 mm). The device measures the movement of the trajectory of the falling rod, impact velocity, spinal cord compression distance, spinal cord compression time, and spinal cord compression rate. After contusion injury, these parameters were examined, and if they were not within the allowable range, the rats were excluded from the study. After wound closure, iodine was applied to the skin. After surgery, the rats were maintained at a warm temperature using a heat pad until they recovered from anesthesia. Subsequently, for at least 7 days, bladder evacuation was performed two times daily, along with the prophylactic administration of cephalosporin (one time per day). During the bladder evacuation, the condition of the wound was checked and iodine was also applied to the skin once daily. Animal use was approved by the National Sun Yat-Sen University Animal Care and Use Committee (Approval No. IACUC-10330).

### Preparation and activation of PRP

Blood was harvested from healthy female Wistar rats through cardiac punctures under anesthesia. The blood was anticoagulated with 3.8% sodium citrate at a blood/citrate ratio of 9:1. Then, the blood was centrifuged at 220 g for 20 min, and the supernatant (containing plasma and buffy coat) was collected and transferred to a new tube for centrifugation at 480 g for 20 min at 4°C. The pellet was extracted and diluted with the supernatant (plasma) to form the final platelet concentration. The platelets were counted using a method from Rees and Ecker. In brief, the diluted PRP was mixed with a solution of 0.2 mL of 40% formaldehyde and 0.1 g of brilliant cresyl blue in 100 mL ultrapure water at a ratio of 1:1, and platelets were counted using hemocytometers. To obtain activated autologous thrombin, blood was collected without anticoagulation, incubated at room temperature until coagulation, and then centrifuged at 3,200 rpm for 3 min. PRP was activated using autologous thrombin and 10% CaCl_2_ and administered by i.t. injection immediately after activation.

### Treatment with PRP

The rats were randomized into the following treatment groups (The volume of i.t. injection in all groups was 10 μL/rat followed by 10 μL of saline): Control group: rats received i.t. injections of normal saline. SCI + vehicle group: rats received i.t. injections of normal saline immediately after SCI and for the following 6 days (7 times in all). SCI + plasma group: rats received i.t. injections of plasma immediately after SCI and after 6 days. SCI + PRP group: rats received i.t. injections of 4.8 × 10^6^ of platelets (activated) immediately after SCI. SCI + PRP-sus: rats received i.t. injections of 4.8 × 10^6^ of platelets (activated) immediately after SCI, and 1.6 × 10^6^ of platelets (activated) for the following 6 days.

### Behavioral analysis

The 21-point Basso-Beattie-Bresnahan (BBB) locomotion scale was used to assess the locomotor behavior recovery after SCI. The BBB scores were documented by independent examiners who were unaware of the experimental groups.

### Tissue processing, staining, and image analysis

The immunofluorescence protocol procedures and the quantification of images were performed as previous described (Chen et al., 2015, 2016). Briefly, under completely anesthetized by isoflurane, the rats (*n* = 4 per each group per time point) were intracardially perfused with ice-cold phosphate-buffered saline (PBS) (pH 7.4) with heparin (0.2 U/mL) and followed by ice-cold 4% paraformaldehyde in PBS. The T8–T12 level of spinal cord was collected. To decrease the variation across following procedures, spinal cords collected at the different time points from different treatment were mounted on the same tissue block by using Tissue-Tek O.C.T. (optimal cutting temperature) compound (Sakura Finetek Inc., Torrance, CA, USA). The tissue block was processed for standard frozen sectioning. The sections (20 μm thick) were air-dried at room temperature, and incubated with blocking buffer (4% normal horse serum diluted in 0.01% Triton X-100 and PBS) for 1 h. The sections were incubated with primary antibodies (Table 1) overnight at 4°C, and then incubated with secondary antibodies (Cy™3-labeled donkey anti-rabbit antibody; Jackson ImmunoResearch Laboratories Inc., West Grove, PA, USA and/or Alexa Fluor 488-labeled chicken anti-mouse antibody; Molecular Probes Inc., Eugene, OR, USA) at room temperature for 1 h.

**Table 1:** Details of antibodies used in this study.

**Antibody**	**Supplier**	**Catalog #**	**Host**
5-HT	ImmunoStar (Hudson, WI, USA)	20079	Goat
Cd11b (OX-42)	Millipore (Bedford, MA, USA)	CBL1512	Mouse
GAP43	Millipore (Bedford, MA, USA)	AB5220	Rabbit
GFAP	Millipore (Bedford, MA, USA)	MAB3402	Mouse
GFAP	GeneTex (Irvine, CA, USA)	GTX61295	Rabbit
ICAM-1	R&D Systems (Minneapolis, MN, USA)	AF583	Goat
Neurofilament	Chemicon (Temecula, CA, USA)	MAB5262	Mouse
PDGF-B	Abcam (Cambridge, MA, USA)	Ab16829	Rabbit
PDGFRβ	Cell signaling (Danvers, MA, USA)	3169	Rabbit
RECA-1	Bio-Rad Laboratories (Hercules, CA, USA)	MCA970	Mouse
vWF	Millipore (Bedford, MA, USA)	AB7356	Rabbit

For eriochrome cyanine (EC) myelin specific staining (*n* = 4 per each group). The sections were incubated with eriochrome cyanine-R reagent (0.16% eriochrome cyanine-R (Sigma-Aldrich St. Louis, MO, USA) and 10% FeCl_3_•6H_2_O (Sigma-Aldrich St. Louis, MO, USA) in 0.5% H_2_SO_4_) for 30 min at room temperature, following by washed with running tap water and differentiated in 1% ammonium hydroxide. The sections were counterstained with neutral red (Sigma-Aldrich St. Louis, MO, USA). The areas of spared white matter (stained as blue color) were calculated by subtracting the cavity areas and gray matter from the total spinal cord sectional areas by using MetaVue Imaging software (Molecular Devices Corporation, Downingtown, PA, USA). The portion of spared white matter was calculated as follows: (spared white matter/total area of spinal cord) × 100. The lesion epicenter was identified by the largest cavity size.

The images were examined using a Leica TCS SP5II microscope (Leica, Wetzlar, Germany) and captured using a SPOT Xplorer Digital camera (Diagnostic Instruments Inc., Sterling Heights, MI, USA). For analysis of immunoreactivity (IR) level, sections located at approximately 400 μm rostral to the lesion epicenter were chose. Four square regions of interest (ROIs, 200 × 200 μm) were placed on each section near the lesion border. The exposure times were the same for all spinal cord sections mounted on the same microscope slide. The MetaVue Imaging software was used to calculate the IR level data (density value/unit area). The IR level data were shown as fold change relative to the control group or the SCI + vehicle group, which was considered to represent a fold change of 1.

To observe blood vessels more clearly, we used confocal image stacks obtained using a Leica TCS SP5II equipped with a Leica hybrid detector (Figures 4B,C). The blood vessel analysis was modified from (Matsushita et al., 2015). For vessel size analysis, four regions of interest (300 × 300 μm) were placed at the gray matter area at 1000 μm rostral to the lesion epicenter. For blood vessel quantification, branched vessels were counted as one vessel. The blood vessel diameter was divided into three categories: 0–7, 8–13, and ≥14 μm.

### Statistical analysis

The data in this study are presented as the mean ± the standard error of the mean (SEM). The neurological outcomes after SCI were analyzed using a two-way repeated-measures analyses of variance (ANOVA) with groups and time points as independent variables, followed by a post-hoc pairwise multiple comparison using the Student–Newman–Keuls method. For immunohistochemical analyses, significant differences between the treatment groups were assessed using one-way ANOVA followed by Student–Newman–Keuls post hoc tests for multiple comparisons. A *P*-value of < 0.05 was considered statistically significant.

## Results

### Effect of i.t. Prp on glial cell in normal spinal cords

**Figure 1:**
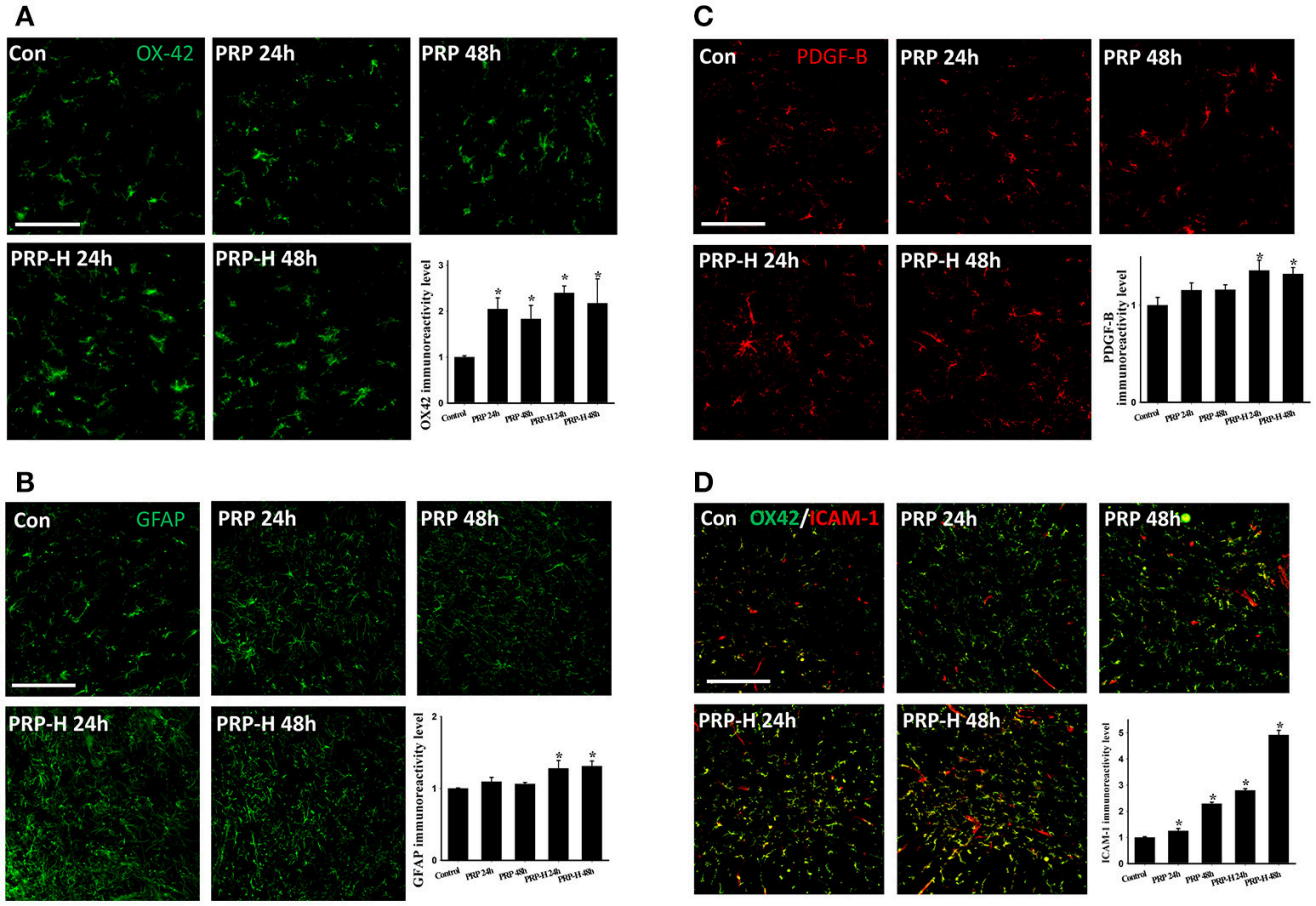
Effect of direct i.t. injection of PRP in normal spinal cords. The spinal cords were harvested 24 or 48 h after two doses of PRP i.t. administration. In the PRP or high dose PRP (PRP-H) treatments, the microglia were activated at both 24 or 48 h after injection **(A)**. However, the astrocytes responded only to a high dose of PRP administration **(B)**. The PDGF-B IR signal was significantly upregulated after a high dose of PRP administration at 24 or 48 h **(C)**. The ICAM-1 IR signal was significantly upregulated after two doses of PRP administration **(D)**. Scale bars = 100 μm (in **A–C**); 200 μm (in **D**). Data are expressed as the mean ± SEM. ^*^P < 0.05, compared with the control group.

According to our review of relevant literature, the effects of i.t. PRP on normal spinal cords remain unclear. Therefore, the normal rats were first given a high and a low dose of i.t. PRP with platelet concentrations of 9.6 × 10^6^ platelets/rat and 4.8 × 10^6^ platelets/rat, respectively. Spinal cords were harvested at 24 or 48 h after PRP injection. We did not observe any neurological defects after two doses of PRP administration within 48 h. The microglia were identified using the OX-42 antibody. After PRP injection, the OX-42 immunoreactivity (IR) signal was significantly upregulated at two doses and two time points after administration (Figure 1A). However, no significant difference was observed between PRP doses and time points. Notably, the astrocytes did not respond to PRP injection in low doses, and the IR signal of the astrocyte specific marker, glial fibrillary acidic protein (GFAP), was upregulated only in spinal cords injected with high doses of PRP (Figure 1B). PDGF-B plays important roles in adult CNS angiogenesis (Cao et al., 2003; Guo et al., 2003); therefore, we examined its effects on normal and injured spinal cords. After PRP injection, the PDGF-B IR signal was significantly upregulated in the high PRP dose group compared with the control group, and the PDGF-B IR signal was not significantly upregulated in the low dose group (Figure 1C). Because PRP may cause inflammation after administration, we used ICAM-1 as a marker to examine the effect of PRP on inflammation induction. The ICAM-1 IR signal was colocalized with the OX-42 IR signal (microglia) (Figure 1D). Some ICMA-1 IR signals were not colocalized with the long tube-shaped microglia. The ICAM-1 IR signal was significantly upregulated in all PRP-injected groups compared with the control group. Moreover, the ICAM-1 IR signal behaved in a dose-dependent manner.

### I.t. PRP-enhanced locomotor recovery after SCI

**Figure 2:**
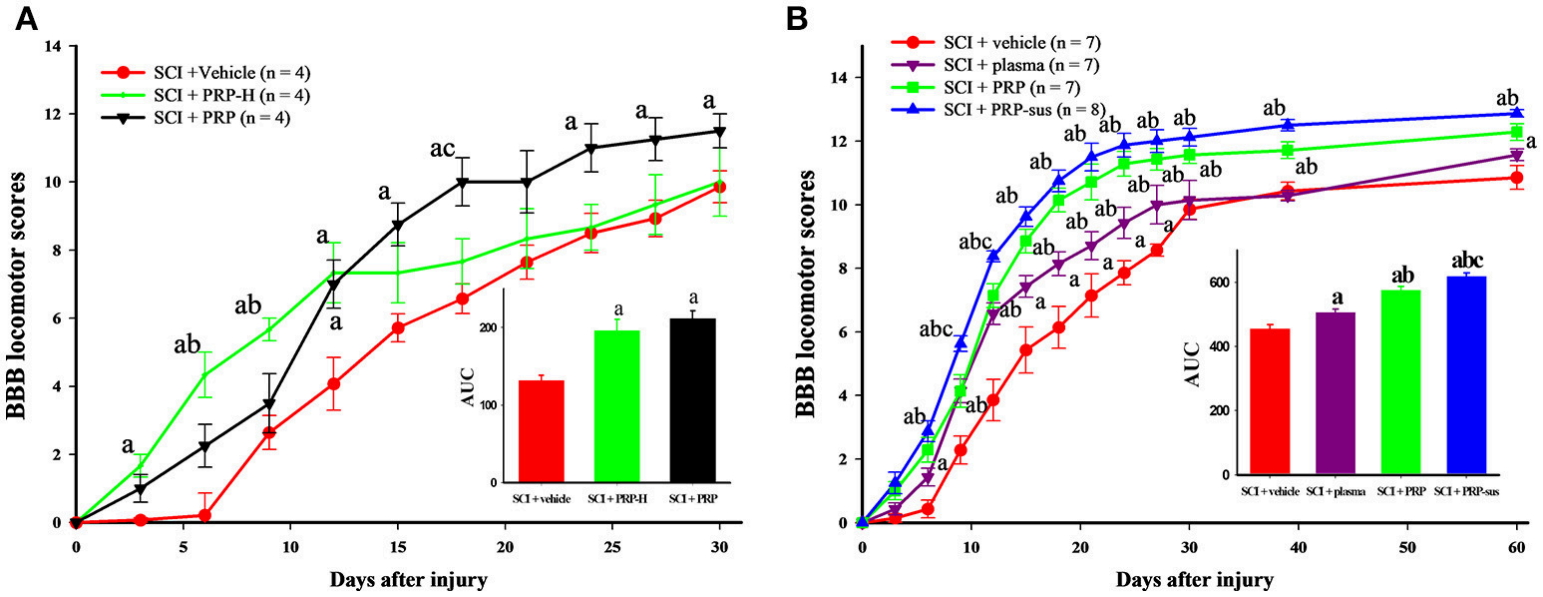
I.t. PRP improved locomotor recovery after SCI. The BBB score was used to evaluate locomotor behavior every 3 days after SCI. In the pilot test, the AUC data suggest that PRP or high-dose PRP treatment improved locomotor recovery after SCI **(A)**. The plasma, PRP, and PRP-sus treatments significantly improved locomotor recovery compared with the vehicle group **(B)** a *P* < 0.05 compared with SCI + vehicle group; b *P* < 0.05 compared with SCI + PRP group; c *P* < 0.05 compared with SCI + PRP-H group. The AUCs of PRP and PRP-sus treatments were significantly higher compared with the plasma group, and the SCI + PRP-sus group had the highest AUC among all groups. Data are expressed as the mean ± SEM. a *P* < 0.05 compared with SCI + vehicle group; b *P* < 0.05 compared with SCI + plasma group; c *P* < 0.05 compared with SCI + PRP group.

The experiment in normal rats suggested that high PRP doses had a more favorable effect in glial cell activation and growth factor release. Therefore, we used the same doses, namely 9.6 × 10^6^ platelets/rat and 4.8 × 10^6^ platelets/rat, in rats with SCI. The rats were randomly divided into three groups after SCI. The SCI + vehicle group received saline, and the SCI + PRP-H and SCI + PRP groups received PRP (9.6 × 10^6^ platelets/rat and 4.8 × 10^6^ platelets/rat; i.t., respectively). Immediately after SCI, each rat in the SCI + vehicle, SCI + PRP-H, and SCI + PRP groups received one i.t. injection. Two doses of PRP exerted the effect of enhanced locomotor behavior after SCI (Figure 2A). Higher doses demonstrated a more favorable locomotor score in the SCI + vehicle and SCI + PRP groups within 12 days after SCI. By contrast, the SCI + PRP group demonstrated a more favorable locomotor score within 12 days after SCI. The data were converted into BBB score–time area under curve (AUC). Clearly, PRP treatment enhanced the recovery behavior in the SCI + PRP group compared with the vehicle and PRP-H groups. Accordingly, we selected a PRP dose of 4.8 × 10^6^ platelets/rat for further experiments.

Based on the aforementioned experiment, we randomly divided the rats into four groups after SCI. The treatments and groupings were as mentioned in the Materials and Methods section. After SCI, the locomotor behavior of rats in the SCI + vehicle group gradually recovered (Figure 2B). Plasma administration significantly enhanced the BBB score on days 14–28 after SCI. The PRP treatment (including one dose and sustained delivery treatments) significantly enhanced the locomotor score on days 12–60 after SCI. At day 60 the rats in the SCI + vehicle group exhibited “occasional weight-supported dorsal stepping” (scores = 9; 1/7), or “occasional weight-supported plantar steps” (scores = 10; 1/7), most of rats exhibited “frequent to consistent weight-supported plantar steps” (scores = 11; 3/7), or “occasional forelimb (FL) – hindlimb (HL) coordination” (scores = 12; 2/7). In the SCI + plasma group most rats exhibited “occasional FL – HL coordination” (scores = 12; 4/7), followed by “consistent weight-supported plantar steps” (scores = 11; 3/7). In the SCI + PRP group most of rats exhibited “frequent FL – HL coordination” (scores = 13; 3/7) or “occasional FL – HL coordination” (scores = 12; 3/7), only one rat had score of 11. In the SCI + PRP-sus group, most rats had score of 13, only one rat had score of 12. The AUC data clearly revealed that PRP treatment enhanced behavior recovery compares with plasma treatment. The PRP-sus treatment group exhibited the most favorable therapeutic effect after SCI among all groups.

### Effect of i.t. PRP on glial cell-related response in injured spinal cords

**Figure 3:**
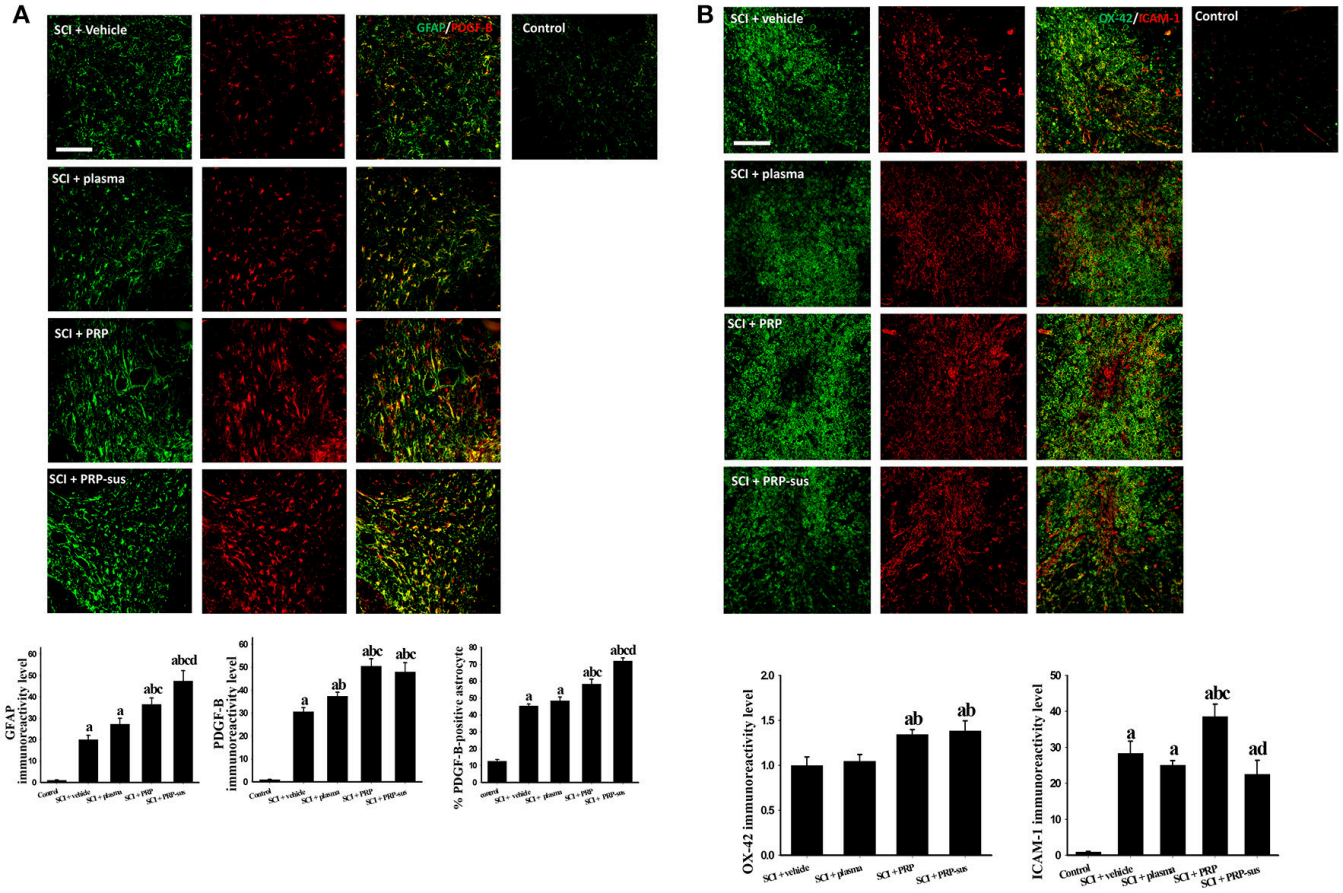
Effect of PRP on SCI on day 7 after SCI. The rats were sacrificed on day 7 after SCI. The PRP and PRP-sus treatments significantly enhanced SCI-induced astrocyte activation and SCI-induced astrocytic PDGF-B expression. The number of PDGF-B-positive astrocytes in the SCI + PRP and SCI + PRP-sus groups was significantly higher than that in the SCI + vehicle or SCI + plasma groups. **(A)**. The PRP treatment significantly enhanced SCI-induced microglia activation **(B)**. Notably, PRP treatment significantly enhanced SCI-induced upregulation of ICAM-1 IR signal. Scale bars = 200 μm. Data are expressed as the mean ± SEM. a *P* < 0.05 compared with control group; b *P* < 0.05 compared with SCI + vehicle group; c *P* < 0.05 compared with SCI + plasma group; d *P* < 0.05 compared with SCI + PRP group.

On day 7 after SCI, four rats from each group were sacrificed to examine the effect of PRP on glial cell (microglia and astrocytes) activation and PDGF-B and ICAM-1 expression. The astrocytes were activated (increased GFAP IR signal) on day 7 after SCI (Figure 3A). No significant difference in GFAP IR signal was obtained between the SCI + vehicle and SCI + plasma groups. However, PRP administration significantly enhanced the GFAP IR signal compared with the SCI + vehicle and SCI + plasma groups. The administration of i.t. PRP significantly upregulated the astrocytic PDGF-B IR signal compared with the SCI + vehicle and SCI + plasma groups after SCI. The number of PDGF-B-positive astrocytes in the SCI + PRP and SCI + PRP-sus groups was significantly higher than that in the SCI + vehicle or SCI + plasma groups. I.t. PRP significantly enhanced PDGF-B expression in astrocytes after SCI. The OX-42 IR signal was significantly upregulated in the SCI + PRP group compared with the SCP + vehicle and SCI + plasma groups after SCI (Figure 3B). No significant difference in the OX-42 IR signal was observed between the SCI + vehicle, SCI + plasma, and SCI + PRP-sus groups. After SCI, the ICAM-1 IR signal was significantly upregulated in all groups compared with the control group. On day 7 after SCI, the ICAM-1 IR signal was also significantly upregulated compared with the control group. Notably, the SCI + PRP group had the highest ICAM-1 IR signal among all groups. No significant difference in the ICAM-1 IR signal was observed between the SCI + vehicle, SCI + plasma, and SCI + PRP-sus groups.

### Effect of PRP on microvessels after SCI and prevention of pericyte disassociation from microvasculature after SCI

**Figure 4:**
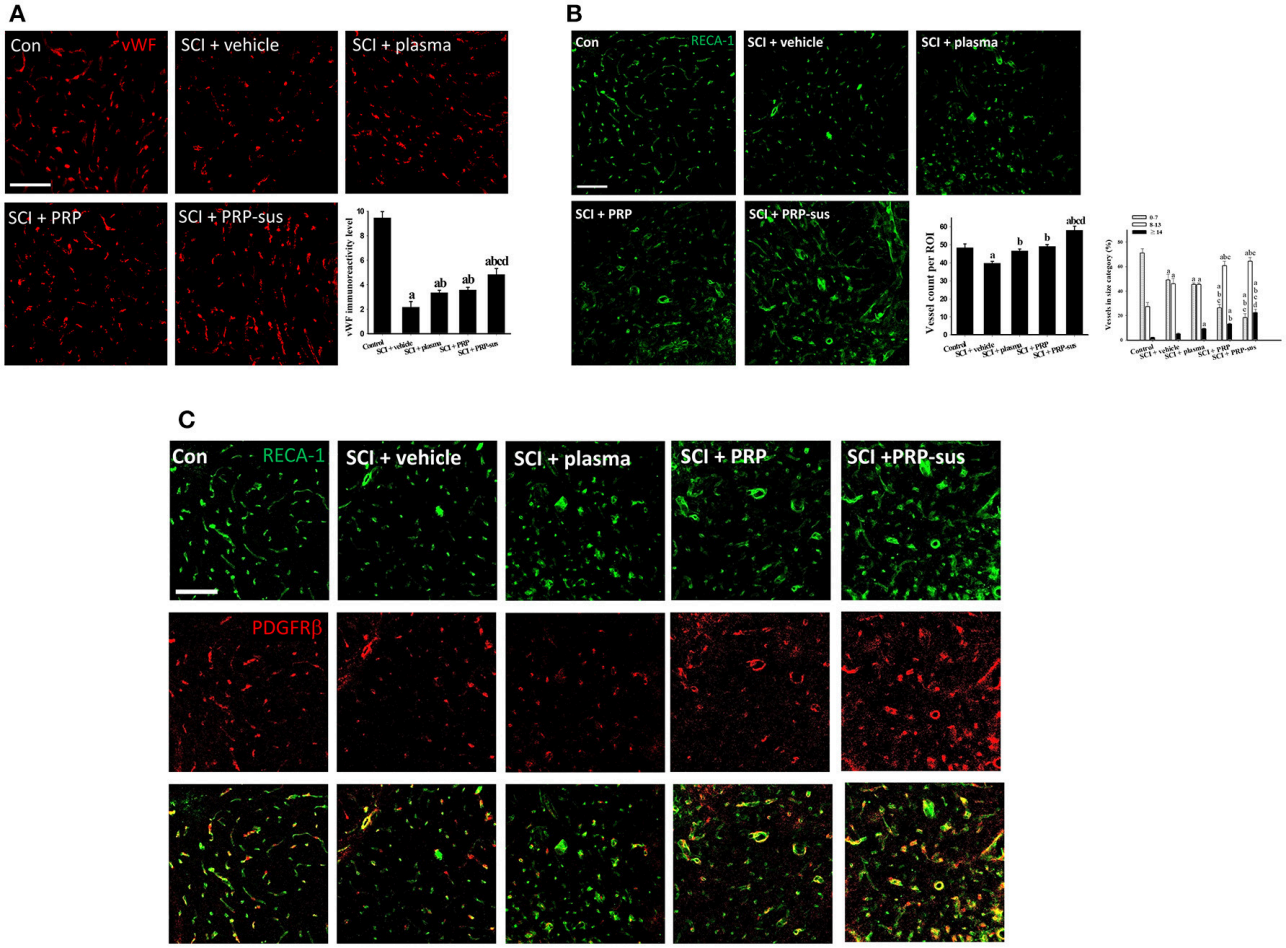
I.t. PRP promoted angiogenesis after SCI. Rats were sacrificed on day 60 after SCI, the cyro-sections of spinal cords were stained with vWF **(A)** and RECA-1 **(B)** antibodies. The vWF IR signal and RECA-1 positive blood vessel count indicate that plasma, PRP, and PRP treatments increased the blood vessel number after SCI. Furthermore, the blood vessels in PRP or PRP-sus treatment had larger diameters compared with vehicle or plasma treatment. The PRP and PRP-sus treatment also increasen in blood vessels associated with pericytes (identified by PDGFRβ antibody; **C**). Scale bars = 100 μm. Data are expressed as the mean ± SEM. a *P* < 0.05 compared with control group; b *P* < 0.05 compared with SCI + vehicle group; c *P* < 0.05 compared with SCI + plasma group; d *P* < 0.05 compared with SCI + PRP group.

The cyro-sections of spinal cords were stained using von Willebrand factor (vWF) (Figure 4A) or rat endothelial cell antigen (RECA-1) (Figure 4B) antibodies. The highest vWF IR signal of all groups was observed in the control group. The spinal cord cyro-sections in the SCI + vehicle, SCI + plasma, SCI + PRP, and SCI + PRP-sus groups were selected at 1,000 μm from the epicenter at 2 months after SCI. After SCI, the length of the vWF IR signal in the vehicle group was shorter than that in the control group. Plasma treatment significantly enhanced the vWF IR signal compared with that of the control group. In addition, the vWF IR signals in the SCI + PRP and SCI + PRP-sus groups were significantly higher than that in the SCI + vehicle group. The shape of the vWF IR signal in the SCI + PRP-sus group was similar to that in the control group. To further examine the effect of PRP on blood vessel number after SCI, we stained the spinal cords with RECA-1 antibody. The number of blood vessels significantly decreased in the vehicle group compared with the control group. Plasma, PRP, and PRP-sus treatments increased the number of vessels after SCI. The SCI + PRP-sus group had the highest number of vessels among all groups. The shape of the RECA-1 IR signal was different after SCI; the long tube-like shape changed to a short circular shape after SCI. Notably, the RECA-1 IR signal in the SCI + PRP-sus group presented a long shape for all treatments. Furthermore, the vessel size was examined using RECA-1 immunofluorescent staining. In normal spinal cords, the size of the majority of vessels was less than 7 μm, followed by 8–13 and ≥14 μm in diameter. After SCI, the vessel size was altered. The number of vessels with diameter < 7 μm decreased, and the numbers of vessels with diameters 8–13 and ≥14 μm increased. PRP and PRP-sus treatment significantly increased the numbers of vessels in the 8–13 and ≥14 μm groups compared with the numbers in the vehicle and plasma groups. To further assess the effect of PRP on pericytes after SCI, the PDGF receptor β (PDGFRβ) antibody was used to identify the pericytes in spinal cord sections. Figure 4C presents a high-magnification version of the image in Figure 4B. In control spinal cords, most RECA-1 (blood vessel) IR signals were colocalized with the PDGFRβ (pericyte) IR signal. In the SCI + vehicle and SCI + plasma groups, some RECA-1 IR signals were not colocalized with the PDGFRβ IR signal. In the SCI + PRP and SCI + PRP-sus treatment groups, most RECA-1 IR signals were colocalized with the PDGFRβ IR signal.

### I.t. PRP enhanced spared white matter after SCI

**Figure 5:**
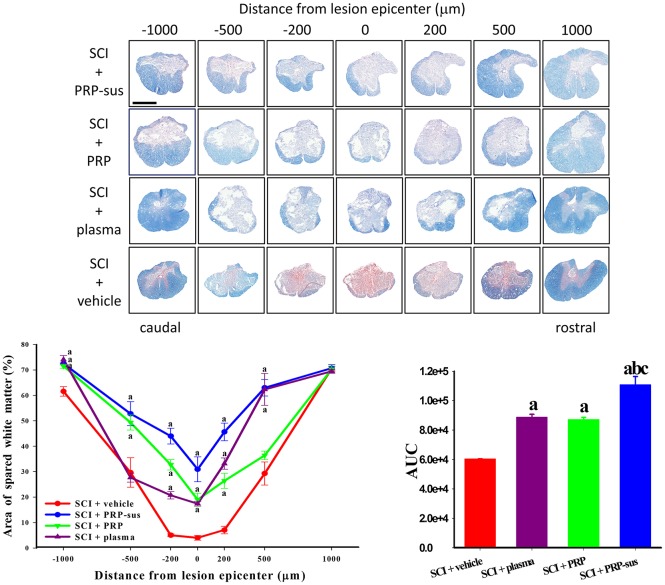
PRP treatment enhanced spared white matter after SCI. The rats were sacrificed on day 60 after SCI, and the spinal cords were sectioned serially. Plasma, PRP, and PRP-sus treatments significantly enhanced the spared white matter compared with vehicle treatment after SCI. The PRP-sus treatment exhibited the highest spared white matter among all treatments. Scale bars = 1 mm. Data are expressed as the mean ± SEM. a *P* < 0.05 compared with SCI + vehicle group; b *P* < 0.05 compared with SCI + plasma group; c *P* < 0.05 compared with SCI + PRP group.

The rats were sacrificed on day 60 after SCI. The continuous spinal cord sections were stained with myelin with EC and counterstained with neutral red (Figure 5). The AUCs of the SCI + plasma, SCI + PRP, and SCI + PRP-sus reveal that these treatments significantly enhanced the spared white matter in these groups compared with the SCI + vehicle group after SCI. However, the AUCs of the plasma and PRP treatment groups exhibited no significant difference. The AUC of the PRP-sus treatment group exhibited a significant difference compared with those of the SCI + vehicle, SCI + plasma, and SCI + PRP groups.

### I.t. PRP enhanced neuronal regeneration after SCI

**Figure 6:**
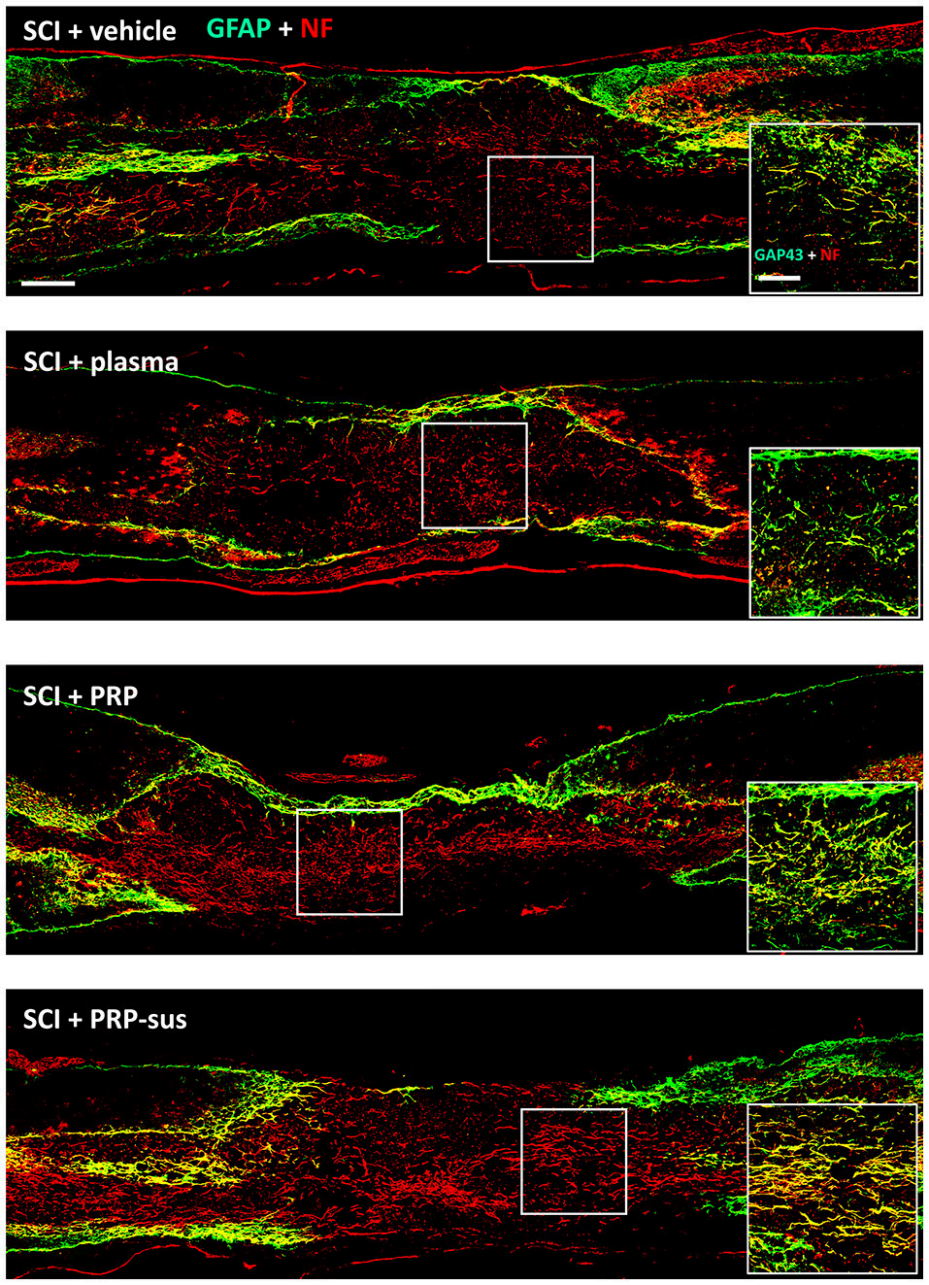
PRP treatment promoted neuroregeneration after SCI. The spinal cords were harvested on day 60 after SCI. The NF IR signals in the PRP and PRP-sus treatments were long and orderly. The NF and GAP-43 IR signal localization suggests that the NFs in the lesion were regenerated after SCI (inset). Scale bars = 500 μm; 200 μm (in inset).

**Figure 7:**
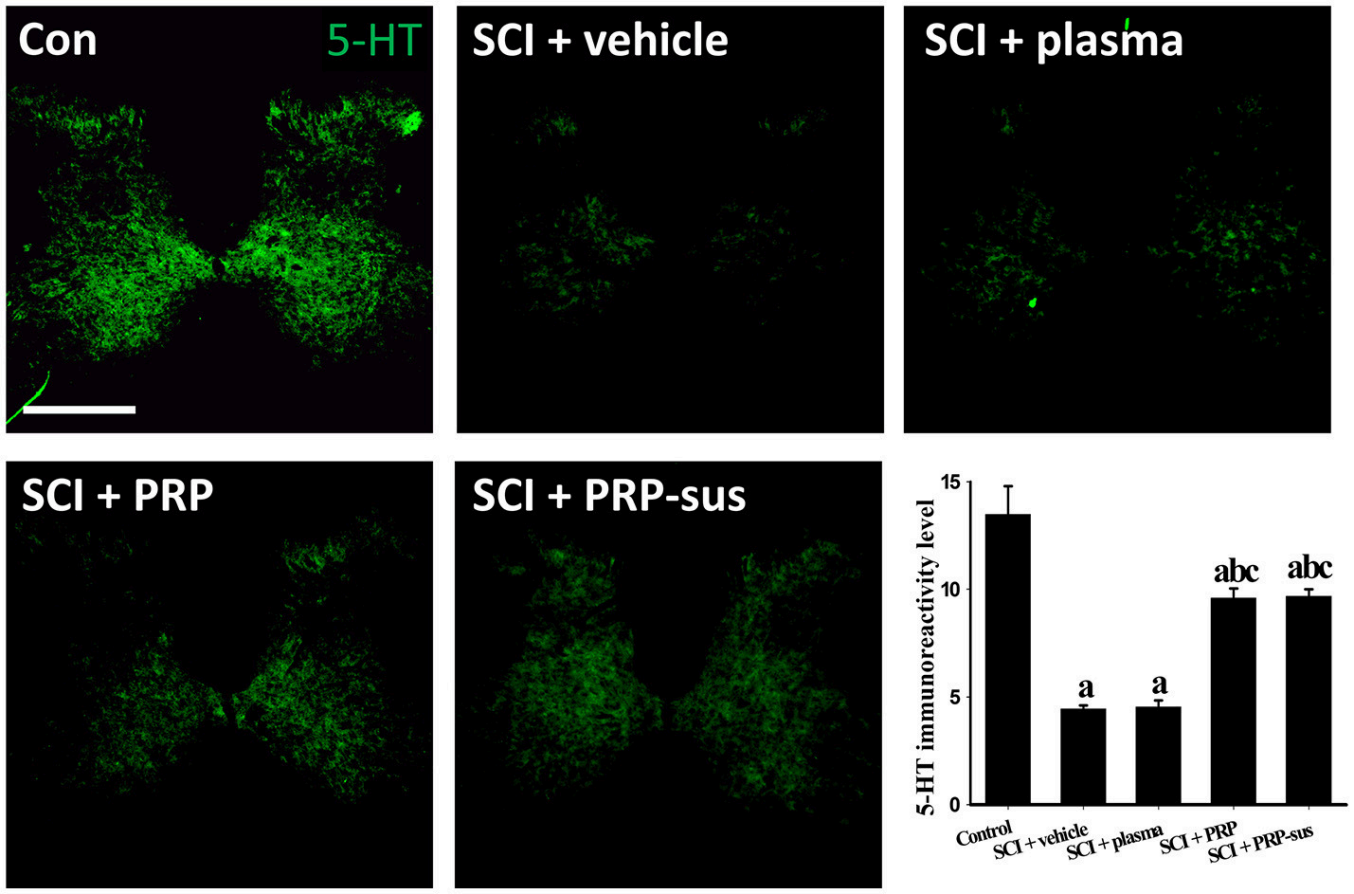
5-HT intensity across lumbar coronal sections. SCI led to a significant decrease in 5HT IR signal compare to control groups. The 5-HT IR signal was significantly higher in the SCI + PRP and SCI + PRP-sus groups than in the SCI + vehicle and SCI + plasma groups. Scale bar = 500 μm. Data are expressed as the mean ± SEM. a *P* < 0.05 compared with control group; b *P* < 0.05 compared with SCI + vehicle group; c *P* < 0.05 compared with SCI + plasma group.

At 2 months after SCI, the rats were sacrificed. The sagittal sections of the spinal cords were stained with GFAP and neurofilaments (NFs) (Figure 6). The immunofluorescence result revealed that the NF IR signals in the SCI + vehicle and SCI + plasma groups were short and nonorderly. However, the NF IR signals in the SCI + PRP and SCI + PRP-sus groups were long and orderly in a particular direction, especially in the SCI + PRP-sus group. To confirm the regeneration of the NF IR signal in the lesion site, we performed tests with growth associated protein 43 (GAP-43), the marker of regenerated axons (White et al., 2008; Li et al., 2011; Pawar et al., 2015) and NF double staining. The result (Figure 6, inset) revealed that the NF IR signal colocalized with the GAP-43 IR signal in all groups, suggesting that the NFs in the lesion site were regenerated and not NFs that had remained from before SCI or NFs that had been spared by SCI. We further analysis on the 5-hydroxytryptamine (5-HT) neurons (Lang et al., 2015; Liu et al., 2017), the enlarged portion of the lumbar spinal cord (L2–L5) of the rats (60 days after SCI) was examined. The 5-HT immunohistochemistry staining and analysis (Figure 7), revealed that SCI decreased the 5-HT IR signal significantly compared with the control group. The 5-HT IR signal was significantly upregulated in the SCI + plasma group compared with the SCI + vehicle group. The PRP treatment enhanced the 5-HT IR signal significantly compared with the SCI + plasma and SCI + vehicle groups. This data confirm our animal behavior result at 60 days after SCI. However, the GAP-43 IR signal was not fully represented neuronal regeneration (Doster et al., 1991; Tuszynski and Steward, 2012), and the neuronal tracing will be performed in a future study.

## Discussion

In the present study, we directly injected i.t. PRP into rat spinal cords and examined the effect of PRP on normal and injured spinal cords. In normal spinal cords, PRP induced microglia and astrocyte activation and PDGF-B and ICAM-1 expression. In the SCI rats, i.t. PRP enhanced the locomotor recovery and spared white matter, promoted angiogenesis and neuronal regeneration, and modulated blood vessel size.

### Possible roles of PRP-induced inflammation after SCI

When normal spinal cords were treated with low doses or high doses of PRP, the microglia were activated. By contrast, the astrocytes were activated only when treated with a high dose of PRP. The same effect was observed for PDGF-B and ICAM-1 expression in normal spinal cords. The PDGF-B IR signal was mainly colocalized with astrocytes in normal or injured spinal cords, and several studies have suggested that the astrocytes express PDGF-B after stimulation (Bethel-Brown et al., 2012; Xu et al., 2016). Therefore, PDGF-B expression in normal or injured spinal cords may be related to astrocyte activation. The inflammatory EC-adhesion molecule, ICAM-1, plays pro-inflammatory roles after SCI (Hausmann, 2003; Trivedi et al., 2006). The adhesion molecules and the vascular adhesion molecule-1 accelerate the interaction between cells such as neutrophils, macrophages, microglia, and endothelium cells. Therefore, the inflammation effect was promoted after SCI. The ICAM-1 immunoreactivity increased with the dose of PRP and time after injection in normal spinal cords, suggesting that i.t. PRP triggered the inflammation. However, the normal rats treated with i.t. PRP did not show any locomotor dysfunction, suggesting that the degree of PRP-induced inflammation was low.

The CD40 ligand (CD40L) and CD40 play roles in immune-related and vascular pathologies (Aloui et al., 2014). The interaction between CD40 and CD40L expression upregulated the ICAM-1 and VCAM-1 expression (Kapur et al., 2015) in endothelial cells, leading to further inflammatory response. Furthermore, microglia were activated by CD40L, and once activated the microglia upregulated CD40 expression and expressed proinflammatory factors, TNF-α, and inducible nitric oxide synthase (Conrad and Dittel, 2011). Thus, the locomotor outcome in the high-dose PRP group was not, as expected, more favorable than that in the low-dose PRP group. Out of all treatment groups, the highest ICAM-1 and OX-42 levels were observed in the SCI + PRP group (Figure 3). Sustained treatment (PRP-sus group) did not affect SCI-induced upregulation of ICAM-1 and OX-42 IR levels. Thus, these results suggest that PRP induced inflammation, and the low degree of inflammation may be a beneficial effect after SCI. Moreover, many studies had suggest platelets play roles in inflammation (Herter et al., 2014; Jenne and Kubes, 2015; Thomas and Storey, 2015). Therefore, the optimum dose and time course of PRP treatment should be examined in further studies.

### Effect of PRP on blood vessels and neuronal regeneration after SCI

Early studies revealed that a biphasic angiogenic response is obtained after SCI. The number of blood vessels decreased for 2 days after SCI, increased on day 4, and declined on day 7 (Imperato-Kalmar et al., 1997; Casella et al., 2002; Whetstone et al., 2003). The angiogenesis after SCI is therefore considered as an endogenous protection reaction. Thus, microvasculature is a therapeutic target for neuroprotection after SCI. The strategies that involve vascular protection and enhanced endogenous angiogenesis after SCI had beneficial effects after SCI (Samantaray et al., 2016; Yu et al., 2016). Another study demonstrated that the angiogenic factors VEGF-A and PDGF-B were downregulated after SCI (Ritz et al., 2010). Thus, upregulating the angiogenesis factors may enhance recovery after SCI. In the present study, PRP treatment enhanced astrocytic PDGF-B expression and SCI-induced ICAM-1. The vWF IR level and vessel number count through RECA-1 staining demonstrated that the PRP-sus group had the highest angiogenesis effect among all groups. Furthermore, the vessel diameter was modulated by PRP treatment after SCI, and the PRP-sus group had the highest number of vessels with diameter >14 μm. These effects may be related to the roles of PDGF-B in adult CNS angiogenesis (Cao et al., 2003; Guo et al., 2003). Both EC and NF immunostaining in the present study evidenced the beneficial effect of PRP-induced angiogenesis after SCI. In addition, studies have shown that PRP promotes angiogenesis through the angiopoietin 1 (Ang1)-Tie2 pathway (Mammoto et al., 2013), and Ang1 also has therapeutic effects after SCI (Han et al., 2010; Herrera et al., 2010). Many studies reported the association between angiogenesis with neurogenesis (Muramatsu et al., 2012; Vissapragada et al., 2014). In the present study, we also show enhanced angiogenesis by PRP administration was associated with increased neurogenesis (Figures 4, 6). Moreover, PRP is rich in angiogenic factors. The effect of other angiogenic factors in the PRP on SCI requires further investigation in future studies.

The blood spinal cord barrier (BSCB) plays similar roles in the CNS as those by the blood–brain barrier. The BSCB breakdown after SCI was initiated through infiltration of immune cells and various neurotoxic molecules (Sweeney et al., 2016) within 5 min after SCI (Maikos and Shreiber, 2007). Therefore, repair of BSCB has become a target for SCI therapies. Pericytes are cells that play an important role in maintaining the blood–brain barrier integrity and angiogenesis through promotion of endothelial cell survival and migration (Armulik et al., 2010). PDGF-B and PDGFRβ are essential for pericyte–endothelial signaling in CNS blood vessel development and stabilization (Lindahl et al., 1997; Gaengel et al., 2009). The sustained PDGF-B-PDGFRβ signaling in the CNS is required for pericyte survival; therefore, PDGFRβ has become the marker for pericytes in the CNS. Dissociation between pericytes and blood vessels was also observed after traumatic brain injury (Zehendner et al., 2015). PDGF and Ang1 are positive regulators of pericyte migration and proliferation (Aguilera and Brekken, 2014). Thus, the increase in blood vessels associated with pericytes on day 60 after SCI may be because of PRP-induced astrocytic PDGF-B expression and Ang1, which is rich in PRP. Overall, the results in the present study revealed that the PRP-sus treatment had the most favorable therapeutic effects compared with plasma and PRP treatment, which may be attributed to angiogenesis.

Although PRP induced inflammation in normal and injured spinal cords, PRP administered at a low dose and with sustained delivery exerted a therapeutic effect (promoted angiogenesis and neuronal regeneration) after SCI. The results of animal behavior recovery, spared white matter, and NF staining also support the hypothesis. In conclusion, sustained PRP treatment for 7 days after SCI may meet the endogenous angiogenesis time course and enhance the endogenous protection reaction after SCI.

### The benefits of using PRP in SCI treatment

It is known that upregulation of growth factor levels exerts neuroprotective effects in SCI. Possible methods of upregulation of growth factors include intravenous injection of recombinant proteins, transplantation of genetically modified cells, and viral gene delivery. These approaches successfully improve functional recovery and enhance axonal regeneration after SCI in animal models. However, they are not necessarily safe for clinical use. For example, recombinant viral vectors are efficient and induce long-term expression of growth factors, but their therapeutic potential is limited by immunogenicity (Peden et al., 2004), and a high risk of insertional mutagenesis that may induce neoplasia (Marshall, 2003). PRP is an autologous substance that is ready to use after short period of centrifugation (total processes are less than 1 h), no special equipment or techniques are needed, and it is not even necessary to preserve PRP. Moreover, PRP had been tested in many clinical settings. These studies demonstrated that PRP is safe and has positive effects. PRP is a non-toxic, autologous material that does not result in an immune response. Autologous PRP administration is non-immunogenic and shows biocompatible properties. Besides, growth factors contain in PRP such as PDGF-AB, PDGF-BB, TGF-β, VEGF-A, and IGF in PRP was both found in rats (van den Dolder et al., 2006; Solchaga et al., 2014) and human (Bertrand-Duchesne et al., 2010; Pallua et al., 2010; Durante et al., 2013). The above suggest growth factors in human or rat PRP may similar. Because PRP is safe and convenient to prepare, we suggest that i.t. PRP will safely enhance intrinsic/endogenous neuroprotective growth factors to treat SCI or other neurodegenerative diseases.

## Author contributions

N-FC, C-SS, W-FC, and Z-HW: conceived and designed the experiments; N-FC, C-SS, C-HC, C-WF, and H-CH: performed the experiments; N-FC, C-SS, C-HC, C-WF, H-CH, K-HT, and S-NY: analyzed the data; N-FC, C-SS, W-FC, and Z-HW: wrote the paper.

### Conflict of interest statement

The authors declare that the research was conducted in the absence of any commercial or financial relationships that could be construed as a potential conflict of interest.
